# Characteristics of Nitrogen Removal and Extracellular Polymeric Substances of a Novel Salt-Tolerant Denitrifying Bacterium, *Pseudomonas* sp. DN-23

**DOI:** 10.3389/fmicb.2020.00335

**Published:** 2020-03-06

**Authors:** Dan Li, Xihong Liang, Chongde Wu

**Affiliations:** ^1^College of Biomass Science and Engineering, Sichuan University, Chengdu, China; ^2^Key Laboratory of Leather Chemistry and Engineering, Ministry of Education, Sichuan University, Chengdu, China

**Keywords:** salt-tolerant denitrifying bacterium, NaCl stress, auto-aggregation performance, extracellular polymeric substances, protein secondary structures

## Abstract

Extracellular polymeric substances (EPS) influence the auto-aggregation performance of cells and play an important role in nitrogen removal during wastewater treatment. In this study, a salt-tolerant aerobic denitrifying bacterium was isolated from tannery wastewater and identified as *Pseudomonas* sp. DN-23. The strain exhibited significant growth and denitrifying performance, with NaCl contents ranging from 0 to 50 g/L, and high antioxidative enzyme activity, especially that of catalase (CAT), was detected under salt stress. Even greater auto-aggregation ability was observed with elevated NaCl content. Extinction-emission matrix (EEM) and Fourier-transform infrared (FTIR) spectrum analyses showed that the main components of EPS were proteins and polysaccharides. The polysaccharide content was almost unaffected by NaCl stress, while the protein content increased with NaCl stress, and the proteins may play a more important role in auto-aggregation. Analysis of the contents of each protein’s secondary structure suggested that β-Sheets increased with increasing NaCl content, which may be related to the increase of auto-aggregation ability in response to NaCl stress. Therefore, NaCl stress increased the auto-aggregation performance by altering the compositions of EPS and the distribution of protein secondary structures. This study provided further insight into the denitrifying performance, and the relationship between aggregation ability and EPS characteristics under NaCl stress.

## Introduction

Ammonium waste streams are massively discharged from landfills along with fish-canning, dyeing, tannery, and chemical plants. Due to the presence of large amounts of salts as chlorides (0–100 g/L), such streams significantly affect the nitrifying-denitrifying performance of various nitrogen removal processes (Wang et al., 2014; Raman and Kanmani, 2016). Numerous studies have reported that low salinity inhibits the enzyme activities of denitrifying bacteria, whereas high salinity causes cell plasmolysis and eventually leads to bacteria death. Moreover, when the cells are cultured in a high-salt environment, the pathways of nitrogen metabolism would be altered, and the expression of denitrifying genes would be diminished (Yu et al., 2015). In addition to its influence on cells, salt also affects the density and amount of dissolved oxygen in wastewater. For example, the salinity would also change the physical and biochemical properties of activated sludge such as structure, hydrophobicity, setting, flocculation, etc. (Bassin et al., 2012).

In recent years, many heterotrophic nitrifying-denitrifying bacteria have been isolated and introduced into wastewater biological treatment processes, such as *Vibrio diabolicus* SF16 (Duan et al., 2015), *Diaphorobacter* sp. PD-7 (Ge et al., 2015), and *Pseudomonas stutzeri* PCN-1 (Zheng et al., 2014). Moreover, many researchers have utilized autochthonous-halophilic bacteria to treat saline wastewater (Campo et al., 2018; Corsino et al., 2018). Among these, two marine bacterial strains, *Bacillus* sp. KGN1 and *Vibrio* sp. KGP1, were used in the form of aerobic granule sludge to treat saline wastewater in a lab-scale sequencing batch reactor system (SBR) (Eom et al., 2018). The results showed that the SBR containing *B*. sp. HGN1 and *V*. sp. KGP1 was more efficient at removing COD, nitrogen, and phosphate. However, little was known about the effect of NaCl stress on the denitrification characteristics of aerobic salt-tolerant denitrifying bacteria.

In addition to the denitrifying characteristics of salt-tolerant denitrifying bacteria, the auto-aggregation performance changes caused by extracellular polymeric substances (EPS) deserve more attention. EPS are made of various compounds such as proteins, polysaccharides, DNA, and humic acids, and the functional groups of EPS play an important role in microbial aggregation, sludge setting, and biofilm formation (Chen et al., 2013b). In addition, it has been reported that EPS contributes to the adsorption and adhesion of metal ions and organic pollutants because of the hydrophobic and hydrophilic regions in their structures (Jia et al., 2016). Microorganisms growing in unfavorable conditions produce additional EPS to protect themselves by balancing osmotic pressure (Tourney et al., 2009; Corsino et al., 2015). Nowadays, many researchers have reported the importance of EPS in forming granules and biofilms. Campo et al. (2018) showed that microorganisms metabolize EPS as an additional carbon source when facing insufficient energy, and a particular EPS metabolism contributed to aerobic granulation in saline wastewater. It was reported that EPS promoted the aggregation of suspended sludge cells by determining the compositions and physicochemical characteristics of EPS, which was ascribed to the hydrophilic cell surface and reduced zeta potentials (Zhang et al., 2014). In addition, many researchers have explored the relationship between the auto-aggregation abilities and EPS characteristics of culturable microorganisms from special environments. *Acinetobacter calcoaceticus*, used for phenol degradation, exhibited excellent auto-aggregation ability because of the secreted EPS, which had the potential to form single cultured granules (Adav and Lee, 2008). Gupta and Thakur (Gupta and Thakur, 2016) isolated a thermotolerant *Bacillus* sp. ISTVK1 from heat-shocked sewage sludge, and this strain produced plenty of EPS during the removal of wastewater contaminants. Moreover, Li et al. (2018) found that the EPS secreted by *Klebsiella* sp. J1 was able to immobilize hexavalent uranium [U(VI)] and mediate partial [U(VI)] reduction in the presence of H_2_. However, few reports have focused on the auto-aggregation performance and EPS characteristics of salt-tolerant denitrifying bacteria.

The purpose of the present study was to explore the effects of NaCl stress on the growth and denitrifying characteristics of a salt-tolerant aerobic denitrifying bacterium. Special attention was given to the auto-aggregation performance and EPS characterizations of the bacterium in response to NaCl stress.

## Materials and Methods

### Media for Bacterial Enrichment and Cultivation

The compositions of enrichment medium (EM) and basic medium (BM) were as follows (per liter): 0.72 g KNO_3_, 5 g sodium acetate, 1 g K_2_HPO_4_, 1 g KH_2_HPO_4_, 0.5 g MgSO_4_, 30 g NaCl, 1 mL trace element solution, pH 7.0. A modified Bromothymol blue (BTB) medium used for the isolation of salt-tolerant aerobic denitrifying bacteria contained the following components (per liter): 0.72 g KNO_3_, 5 g sodium acetate, 1 g K_2_HPO_4_, 1 g KH_2_HPO_4_, 0.5 g MgSO_4_, 30 g NaCl, 1 mL of trace element solution, 20 g agar, 1 mL BTB solution (1% in ethanol), pH 7.0. The denitrification medium (DM) used for studying the denitrifying characteristics of salt-tolerant bacteria was as follows (per liter): 0.72 g KNO_3_, 8.2 g sodium acetate, 1 g K_2_HPO_4_, 1 g KH_2_HPO_4_, 0.5 g MgSO_4_, 1 mL of trace element solution, pH 8.0. NaCl was added as needed (0, 30, or 50 g/L). The composition of the trace element solution was as follows (per liter): 50 g EDTA, 2.2 g ZnSO_4_⋅7H_2_O, 5.5 g CaCl_2_⋅H_2_O, 2.06 g MnCl_2_, 5 g FeSO_4_⋅7H_2_O, 1.1 g (NH_4_)_6_Mo_7_O_24_⋅4H_2_O, 1.57 g CuSO_4_⋅5H_2_O, 1.61 g CoCl_2_⋅6H_2_O, pH 7.0.

### Isolation and Identification of a Salt-Tolerant Aerobic Denitrifier

10 mL of sludge sample, collected from a sequencing batch reactor in Ruixing Leather Industry Co., Ltd., Zhejiang, China, was transferred to a 250 mL Erlenmeyer flask containing 90 mL of sterilized enrichment medium. After incubating for 48 h (150 rpm, 30°C), the homogeneous bacterial suspensions (10 mL) were transferred to another 90 mL of fresh EM and incubated under the same conditions as above. This procedure was repeated three times. After diluting them by gradient, the bacterial suspensions were spread on the BTB medium plates. The plates were incubated at 30°C for a few days until visible blue colonies were formed. Then, separate colonies were purified via repeated streaking on BTB medium plates. Pure isolates were obtained and their denitrifying ability was tested individually in BM. The isolate with the greatest denitrifying rate was suspended in 30% glycerol solution at −80°C for long-term storage.

To identify the salt-tolerant denitrifying bacterium, total bacterial DNA was extracted using the EZ-10 Column Genomic (Sangon, Shanghai, China). The 16S rDNA gene was amplified by PCR using the universal bacterial primers 5′-AGAGTTTGATCCTGGCTAG and 5′-TACGGTTACCTTGTT ACGACTT, and sequenced by Sangon Biotech Co., Ltd. The sequence was analyzed and compared in the Genbank using BLAST^1^. Finally, a neighbor-joining tree was constructed in MEGA7.

### Optimization of the Cultivation Conditions for Aerobic Denitrification

The growth and aerobic denitrifying characteristics of the salt-tolerant bacterium were conducted under varied culture conditions, including carbon source, C/N ratio, pH, and temperature. In the carbon source experiments, glucose, sodium pyruvate, sodium citrate, and sodium succinate were used in the BM instead of sodium acetate. As for the C/N ratio, different C/N ratios (3, 6, 12, 18, and 24) were obtained by adjusting the sodium acetate content. To determine the effects of pH and temperature on the denitrifying capabilities of the isolate, the initial pH was adjusted to 6, 7, 8, 9, 10, or 11, and the temperature was set at 10, 20, 30, or 37°C. All of the above experiments were conducted with 1% (v/v) inoculation size in 100 mL of sterile medium, in triplicate. The cells were cultivated for 48 h (30°C, 150 rpm) in shake flasks, and samples were harvested to determine the OD_600_ and the concentration of nitrate.

### Effects of NaCl Stress on the Aerobic Denitrification of the Isolate

To evaluate the effects of NaCl stress on the aerobic denitrification characteristics, 1 mL of the pre-cultured isolate was inoculated into 100 mL of sterile DM containing different concentrations of NaCl (0, 30, or 50 g/L). The isolate in the Erlenmeyer flask was aerobically cultured in a shaker (150 rpm) at 30°C. The samples were harvested from the DM periodically by centrifugation (10000 rpm, 5 min). Then, the contents of TN, NO_3_^–^-N, NO_2_^–^-N, and cell optical density (OD_600_) were measured. Each treatment was conducted in triplicate.

### Determination of Antioxidant Enzyme Activities

The strain was cultivated to logarithmic metaphase and the cells were collected by centrifugation (10000 rpm, 4°C) for 5 min, washed two times with ultrapure water, and then resuspended in ultrapure water. The cells were lysed by ultrasonication on ice (20 kHz, 15 min) and then centrifuged (10000 rpm, 10 min, 4°C) to obtain cell extracts as a crude enzyme. Assay Kits (Jiancheng, Nanjing, China) were used to determine the activities of superoxide dismutase (SOD), catalase (CAT), and peroxidase (POD). Protein concentration was determined by using the Bradford Protein Assay Kit (Biyuntian, Haimen, China) (Bin et al., 2008). Enzyme specific activity (U/mg) was defined as the amount of enzyme that transformed 1 μmol substrate per minute by the amount of milligram protein.

### Flocculating Test and the Auto-Aggregation Ability of the Isolate

The flocculating rate of the isolate cultivated in media with different NaCl concentrations (0, 30, and 50 g/L) was determined according to the method reported by Huang et al. (2005). In general, 0.4 g kaolin, 5 mL 1% CaCl_2_, and 93 mL ultrapure water were put into a 200-mL beaker. Then, 2 mL of fermentation broth was added to the beaker. The liquid was agitated for 2 min with a magnetic stirrer, transferred to a 100-mL cylinder, and then remained static for 5 min. The absorbance of the supernatant and control solution (ultrapure water was used instead of fermentation broth) was measured at 550 nm as OD_550_ and OD_550__,control_. All of the above assays were conducted in triplicate. The flocculation rate was defined and calculated by the following Eq. 1 (Zhang et al., 2014):

(1) Flocculatingefficiency(%)=(OD-550,controlOD)550/OD×550,control100

To investigate the auto-aggregation ability, cells cultivated to logarithmic metaphase in DM containing NaCl (0, 30, or 50 g/L) were collected by centrifugation (10000 g, 4°C) for 5 min, washed three times by ultrapure water, and resuspended in the same water. The auto-aggregation rate of cells was determined according to the method reported by Zhang et al. (2014). The initial OD_600_ of the cell suspension was adjusted to 0.6, and 5-mL suspensions were transferred into a test tube. Subsequently, the test tube was left standing for 1, 2, 3, 4, and 5 h, and the OD corresponding to cells at the upper part of the cuvette was measured at 600 nm (A_*t*_) (*t* = 1, 2, 3, 4, 5 h). The auto-aggregation rate (At) of microbial cells was calculated by Eq. (2) (Sheng and Yu, 2006).

(2) Auto-aggregationrate(%)=(1-A/tA)0×100%

Here A_0_ is the OD_600_ at time 0, namely initial OD_600_.

### Zeta Potential

The zeta potentials of cells in response to NaCl stress (0, 30, or 50 g/L) were determined using Zetasizer Nano ZS90 (Malvern Instruments, United Kingdom) according to previous methods (Bernstein et al., 2011). Briefly, the OD_546_ of the collected cells in ultrapure water was adjusted to 0.1 and the zeta potentials were measured by bacterial electrophoretic mobility (Bernstein et al., 2011).

### Extracellular Polymeric Substances (EPS) Extraction

The EPS was extracted according to a modified cation exchange resin (CER) method developed by Liang et al. (2010) Cells cultivated in the DM with NaCl concentrations of 0, 30, and 50 g/L were collected at logarithmic metaphase. Then, 40 mL of each cell suspension was harvested by centrifugation (5000 rpm, 5 min, 4°C), washed three times with ultrapure water, and resuspended to its initial volume with ultrapure water. The OD_600_ of different samples was adjusted to be the same, and then the suspensions were transferred into 250-mL flasks with CER (70 g/g dry cells) added. The flasks were shaken in a thermostat at 150 rpm for 1 h at 4°C and left standing for 3 min to settle the CER. Subsequently, the suspension was centrifuged for 15 min (5000 rpm, 4°C), filtered through a 0.45-μm filter, and stored at −20°C for further analysis. The dry weight of cells was measured according to Standard Methods. The concentration of polysaccharides was determined by anthrone colorimetry (Raunkjær et al., 1994). The concentration of proteins was determined according to the method described in section “Determination of Antioxidant Enzyme Activities.”

### Three-Dimensional Extinction-Emission Matrix (3D-EEM) Spectrum of EPS

The 3D-EEM of EPS was identified using a luminescence spectrophotometer (F-7000 FL, Hitachi, Japan). The EEM spectra of EPS were gathered with subsequent scanning emission (Em) spectra from 250 to 500 nm by varying the excitation (Ex) wavelength from 200 to 450 nm. Both excitation and emission slits were maintained at 5 nm, and the scanning speed was set at 1200 nm min^–1^ for all measurements. Software Origin 9.1 was employed to process the 3D-EEM data. The EEM spectra were plotted as the elliptical shape of contours, whereby the *X*-axis represented the emission spectra from 250 to 500 nm, while the *Y*-axis represented the excitation spectra from 200 to 450 nm.

### Fourier-Transform Infrared (FT-IR) Analysis of the EPS

The FT-IR spectrum was used to analyze the EPS components. The EPS extracts were freeze-dried in a refrigerator for 48 h at −70°C, and the samples were mixed with KBr in an agate grinder at a ratio of 1:100. Then, the homogenized powders were molded into a disk. The FT-IR spectra were recorded in the 4500–400 cm^–1^ region using FT-IR spectrophotometer 510FT (Rayleigh Analytical Instrument Corporation, Beijing). Date derivation was performed with Origin 9.1 software.

### The Measure of Protein Secondary Structure in EPS

Circular dichroism (CD) spectroscopy can be used to analyze the secondary structure of proteins in solution (Perez-Iratxeta and Andrade-Navarro, 2008). The protein concentration of extracted EPS was diluted to 1.0 mg/L. A circular dichroism spectrometer (J715, JASCO, Japan) was used to obtain CD spectra in a 1.00 cm path-length quartz cuvette. The far-UV CD spectrum was corded at the 0.1-nm interval, and the time constant was 3.0 s. The CD spectrum was analyzed and calculated by the self-consistent method SELECON3 (Sreerama and Woody, 2000).

### Analytical Methods

The growth of the isolate was measured at 600 nm (OD_600_) using a Spectrophotometer (UV-1901, Puxi, Beijing). The cell dry weight was obtained by drying at 105°C. The nitrite was measured by N-(1-naphthalene)-diaminoethane ultraviolet spectroscopy at 540 nm (Clesceri et al., 2005). The concentration of total nitrogen (TN) was measured by phenol disulfonic acid ultraviolet spectroscopy, and the nitrate concentration was determined by the phenol disulfonic acid method (APHA, 1998).

## Results

### Isolation and Identification

40 purified strains were tested for their denitrifying ability in a high-salt environment (NaCl 30 g/L). One isolate, named DN-23, demonstrated the greatest nitrate removal ability in BM. The nucleotide sequence of the strain DN-23 was submitted to GenBank nucleotide sequence databases. The blast results suggested that the strain DN-23 showed the greatest similarity (99%) to *Pseudomonas balearica* DSM. A neighbor-joining phylogenetic tree of the isolate was constructed in MEGA 7 based on the nucleotide sequence (Supplementary Figure S1). Based on the result of the 16S rDNA gene sequence, the isolate was named *Pseudomonas* sp. DN-23, and deposited in the China Center for Type Culture Collection (CCTCC: M2018290).

### Denitrification Characteristics of Strain DN-23 Under Various Conditions

**FIGURE 1:**
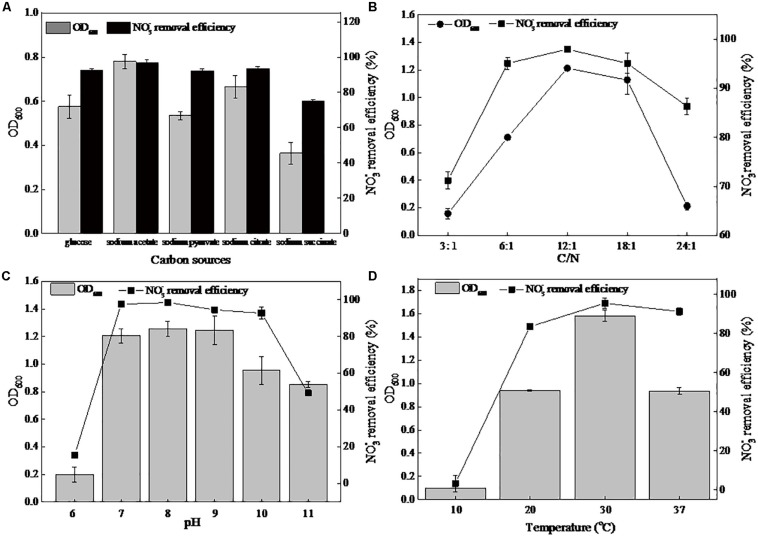
Effects of different factors on the growth and nitrate removal of strain DN-23 in a high-salt environment (30 g/L NaCl). **(A)**: carbon source, **(B)**: C/N, **(C)**: pH, **(D)**: temperature. The biomass and nitrate removal rate were determined after 48 h of culture. Values are means ± SD (error bars) for three replicates.

The carbon source, serving as an energy and electron source, has long been considered to be an important factor for the growth and nitrate removal ability of denitrifiers. In the present study, glucose, sodium acetate, sodium pyruvate, sodium citrate, and sodium succinate were used as the sole carbon source in the DM (Figure 1A). Among these five carbon sources, the maximum biomass (0.77) and the greatest denitrification rate (96.83%) were observed when using sodium acetate. When cultivated in the other four carbon sources, the strain DN-23 had lower growth and denitrification rates (75.13–93.31%). Thus, sodium acetate was employed in subsequent experiments. The effects of C/N on the cell growth and denitrifying rate were explored (Figure 1B). The biomass and nitrate removal efficiency increased with the C/N ratio, from 3:1 to 12:1. The peak of OD_600_ and denitrification rate were 1.21 and 98.03%, respectively, occurring at C/N 12:1. Thus, in the present study, C/N 12:1 was used in subsequent experiments.

The cellular growth and denitrification rates at different pHs are shown in Figure 1C. The strain DN-23 had efficient denitrification abilities at initial pHs of 7–10, namely neutral or slightly alkaline environments. The greatest removal rate of 98.59% and the maximum biomass 1.25 were observed at an initial pH of 8. Figure 1D showed the effects of temperature on the biomass and denitrification ability of strain DN-23. The optimal culture temperature was 30°C, with a maximum OD_600_ of 1.58 and a maximum denitrification rate of 95.53%. The strain was nearly unable to grow at 10°C. As the temperature increased to 37°C, the OD_600_ and denitrification rate reduced to 0.93 and 91.38%, respectively.

### Effects of NaCl on Denitrification Characteristics of DN-23

**FIGURE 2:**
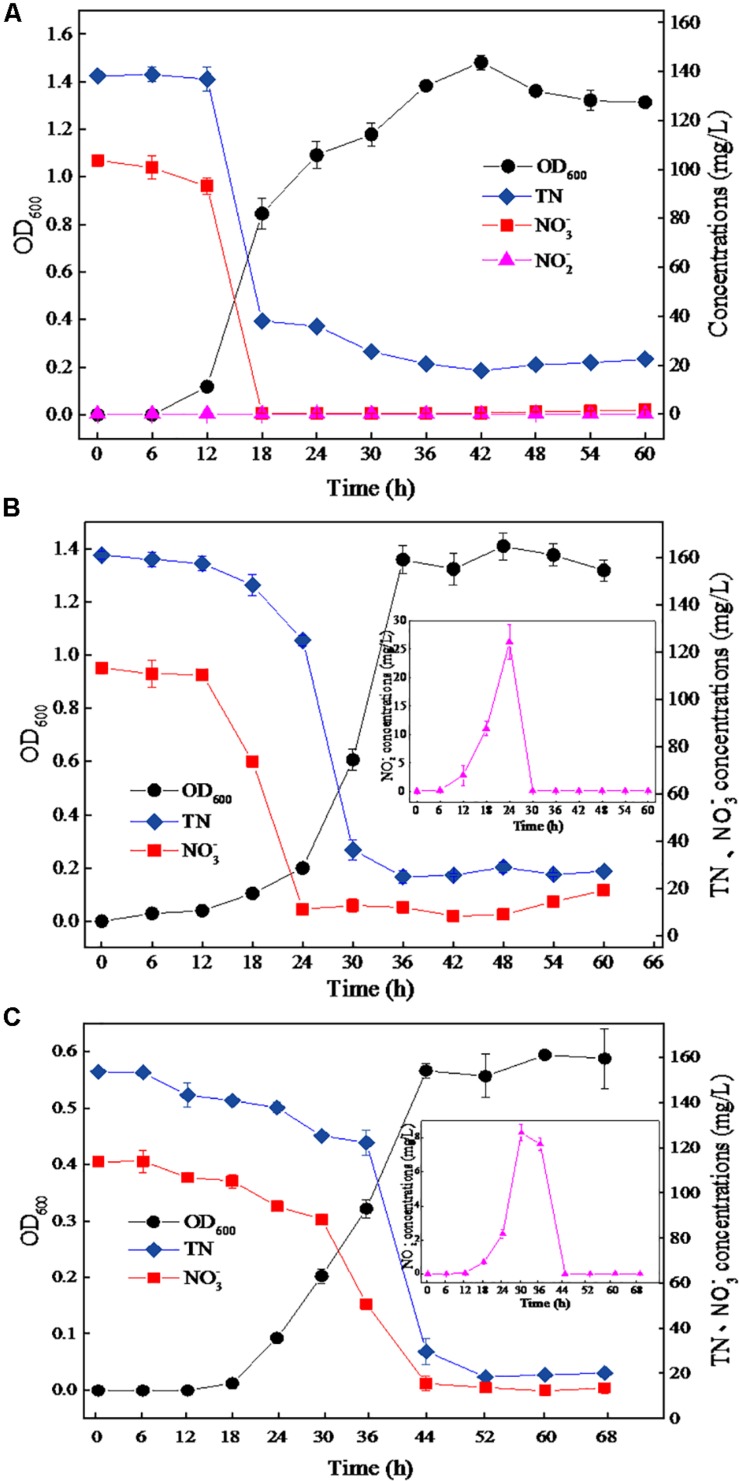
The growth and aerobic denitrification characteristics of strain TN-10 cultivated in DM with different NaCl concentrations **(A**: 0 g/L NaCl, **B:** 30 g/L NaCl, **C:** 50 g/L NaCl). Samples were collected from the DM, and OD_600_, TN, NO_3_^–^-N and NO_2_^–^-N were determined at different time intervals. Values are means ± SD (error bars) for three replicates.

The strain DN-23 was cultivated in DM containing different NaCl concentrations, and the time courses of OD_600_, TN, NO_3_^–^-N, and NO_2_^–^-N were measured (Figure 2). Figure 2A shows the growth and denitrification characteristics of strain DN-23 in the absence of NaCl. The cell growth increased rapidly from 4 h and reached a peak of OD_600_ 1.48 at 42 h, with biomass declining thereafter. As for the nitrate level (103.78 mg/L initial NO_3_^–^-N), it declined rapidly after 12 h and was nearly undetectable after 18 h. The maximum nitrate removal rate was 15.50 mg/L/h, occurring between 12 and 18 h. In addition, the variation tendency of TN concentration corresponded with that of nitrate concentration. The TN concentration decreased rapidly from 12 h onward and remained nearly constant (around 20 mg/L) after 36 h. Nitrite was undetectable during the entire range of the denitrifying process.

Figure 2B shows the growth and denitrification characteristics of strain DN-23 in an environment of 30 g/L NaCl. The cells grew rapidly from 18 h onward, and remained constant after 36 h with an OD_600_ of 1.31–1.40. The nitrate showed a significant decrease from 12–24 h and then remained stable at 11.37 mg/L, and the maximum denitrification rate was 8.26 mg/L/h. The TN also decreased significantly between 12 and 36 h and then held constant at 25.12 mg/L. The results showed that the denitrification rate under stress conditions of 30 g/L NaCl was slightly inhibited compared to 0 g/L NaCl. Additionally, it was noteworthy that NO_2_^–^-N, the metabolite of NO_3_^–^-N, was produced from 6 h onward, and its concentration reached a peak of 26.25 mg/L at 24 h, while nitrite was barely detectable after 30 h.

Figure 2C shows that the strain DN-23 was obviously inhibited under the condition of NaCl 50 g/L. The cells entered into a stable phase at 44 h with OD_600_ 0.56, and the biomass cultivated in the DM with 50 g/L NaCl was significantly greater than that in 0 g/L and 30 g/L NaCl. The nitrate concentration was nearly constant after 44 h with an 86.37% denitrification rate. The TN concentration curve of was consistent with that of nitrate concentration. In addition, nitrite was also formed during the denitrification process, and the peak concentration reached 8.30 mg/L at 30 h. The nitrite was converted completely after 44 h, which was related to the variation of nitrate. Overall, the growth and denitrification performances of strain DN-23 were significantly inhibited under high-salt conditions.

### Effect of NaCl on Antioxidative Enzyme Activity

**FIGURE 3:**
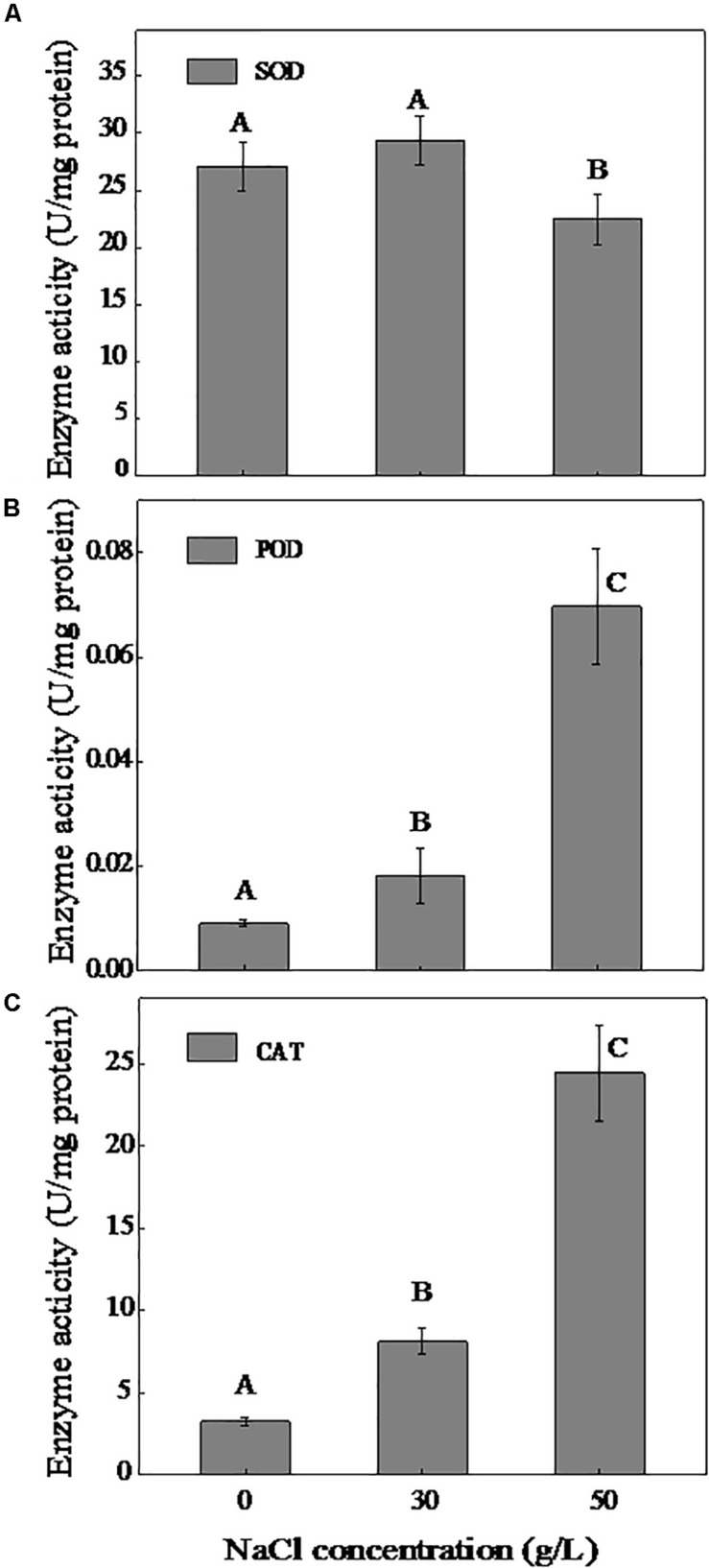
Effect of NaCl on the activities of SOD **(A)**, POD **(B)**, and CAT **(C)**. The cells were cultivated in the DM and collected at logarithmic metaphase (0 g/L NaCl, 24 h; 30 g/L NaCl, 30 h, and 50 g/L NaCl, 36 h). Different uppercase letters are used to indicate the significant differences of enzyme activity at *p* < 0.05 (*n* = 3). Values are means ± SD (error bars) for three replicates.

In the present study, we measured the activities of the main antioxidative enzymes SOD, POD, and CAT. As shown in Figure 3, the SOD activity increased slightly in the presence of NaCl (0–30 g/L) and decreased at a NaCl concentration of 50 g/L. Although the POD activity significantly increased with increasing NaCl concentration, the activity was at a very low level (0.009–0.069 U/mg protein). In addition, the activity of CAT increased considerably with the increase of NaCl content, and a 7.54-fold greater activity of CAT was measured under 50 g/L NaCl compared to 0 g/L NaCl.

### Analysis of Flocculating Efficiency, Zeta Potential, and Auto-Aggregation Rate

**FIGURE 4:**
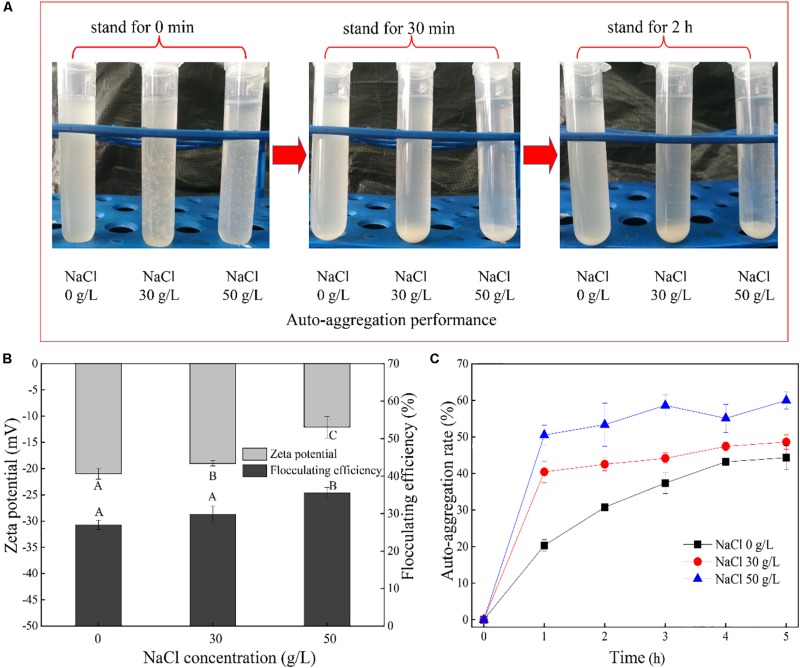
Auto-aggregation characteristics of cells. **(A)**: auto-aggregation performance of cells, **(B)**: flocculating efficiency in kaolin suspension and zeta potential of the cell surface. **(C)**: auto-aggregation rate of strain DN-23. The cells were collected at logarithmic metaphase from the DM with different NaCl concentrations. Different uppercase letters indicate the significant differences of enzyme activity at *p* < 0.05 (*n* = 3). Values are given as mean ± SD (error bars) for three replicates.

Figure 4 shows the flocculation properties of strain DN-23 in response to NaCl stress. As shown in Figure 4A, the cells quickly settled into the bottom of the centrifuge tube. Samples collected from the DM with greater NaCl concentrations had more flocculation in the bottom of the tube after standing. The zeta potential, flocculating efficiency, and auto-aggregation rates were then measured to quantitatively describe the aggregation performances. Zeta potential can be used to describe the electrostatic interactions between colloidal particles, which are important indicators of the stability of the colloidal system (Xu et al., 2014). In addition, the zeta potential is important for adsorption as it can indicate interactions between particles (Zadaka et al., 2010). The more negative the zeta potential, the greater the stability of particle interactions (Tong et al., 2011). Figure 4B shows the flocculating rate in the kaolin suspension and the zeta potential of the cell surface. Obviously, the zeta potentials of cells cultivated in the medium with 0 g/L NaCl were more negative than in media with 30 and 50 g/L NaCl, indicating that the cells were more stable and the flocculating efficiency was lower. Meanwhile, it was noted that the flocculating efficiency increased from 27.01% (NaCl 0 g/L) to 35.56% (NaCl 50 g/L), which was consistent with the results of the zeta potentials. We further investigated the variation of the auto-aggregation rate over time (Figure 4C). The results showed that the strain DN-23 exhibited a greater auto-aggregation ability under high-salt stress. It seems that the auto-aggregation rate reached equilibrium after 3 h, and the auto-aggregation rates were 44.34% (NaCl 0 g/L), 48.62% (NaCl 30 g/L), and 59.99% (NaCl 50 g/L), respectively, after standing for 5 h.

### EPS Characterization by 3D-EEM Fluorescence

**FIGURE 5:**
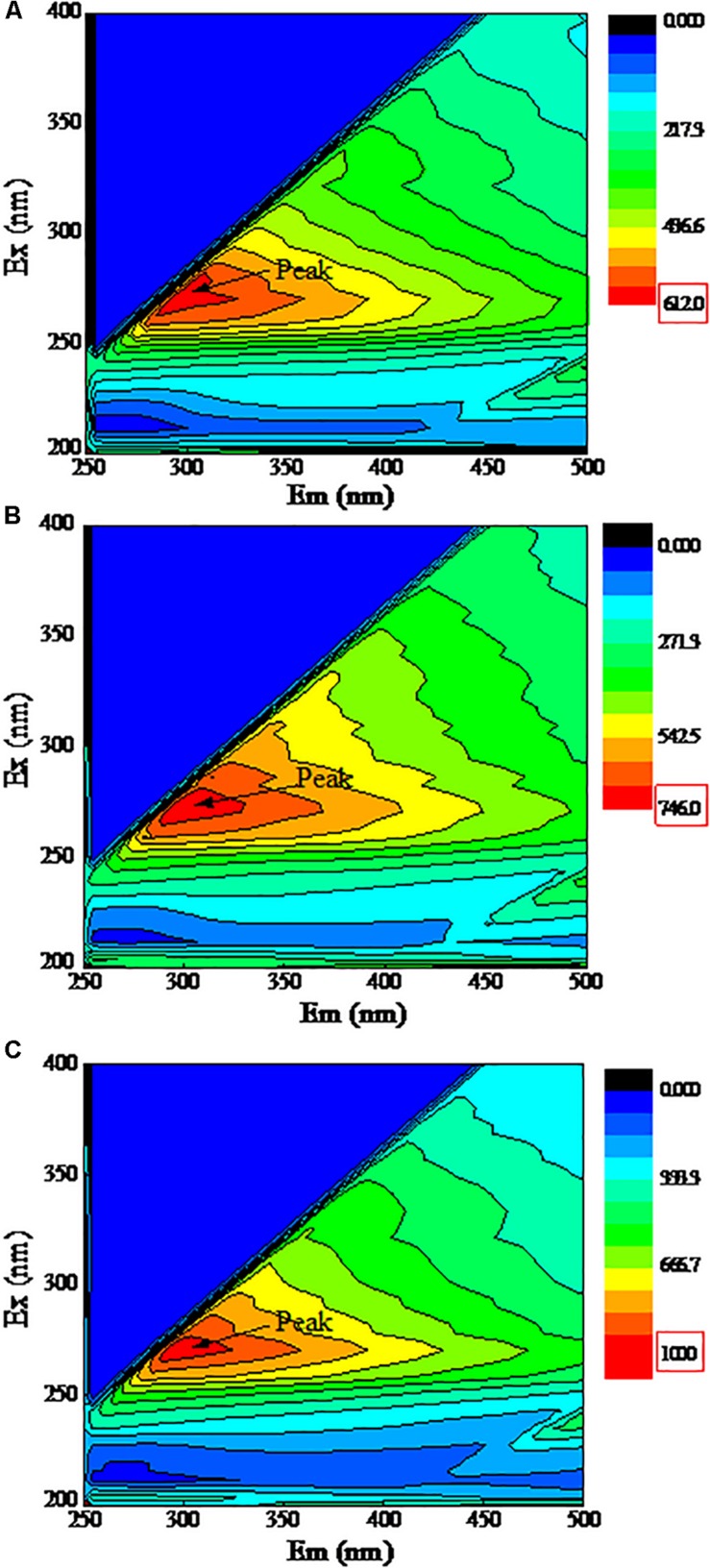
3D-EEM fluorescence spectra of EPS. The *X*-axis represents the emission spectra, *Y*-axis represents the excitation spectra. Cells were cultivated at DM with different NaCl stress, and the samples were collected at logarithmic metaphase. **(A)**: 0 g/L NaCl, **(B)**: 30 g/L NaCl, **(C)**: 50 g/L NaCl.

Figure 5 shows the 3D-EEM fluorescence of EPS extracted from strain DN-23. As shown in Figure 5, only one peak was clearly observed for all samples. Peaks are located at the excitation/emission wavelengths (Ex/Em) 285/290-295, which are all assigned to protein-like substances (Hudson et al., 2008). The fluorescence of peaks implies the presence of aromatic amino acids such as tryptophan or tyrosine. In the present study, all peak locations were close, indicating that the EPS components of all samples were similar. Analysis of the peak intensities showed that the intensity increased with the NaCl concentration in DM.

### FT-IR Spectra of EPS

**FIGURE 6:**
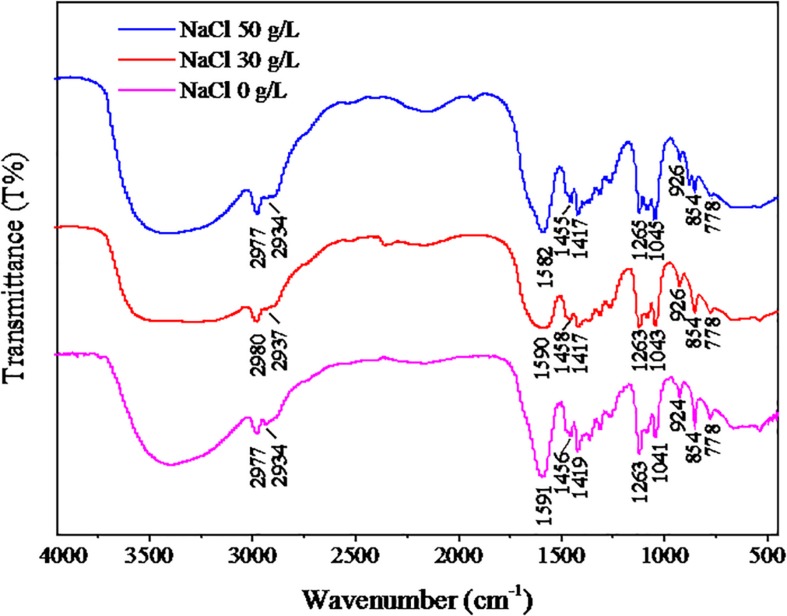
FT-IR results of EPS. The strain was cultivated in the DM with different NaCl concentrations (0, 30, or 50 g/L), and the cells were collected at logarithmic metaphase. The EPS of cells were extracted for FT-IR analysis.

The FT-IR spectra of EPS from the three samples are shown in Figure 6. The results of FT-IR were assigned and compared according to previous literature (Liang et al., 2010). The bands at 2934–2980 cm^–1^ were assigned to the stretching vibration of the C-H group. The adsorption bands at 1582–1591 cm^–1^ were associated with the stretching vibration of C-N in proteins. The bands at 1455–1458 cm^–1^ were related to the C-H deformation in >CH_2_ of proteins. The bands near 1400 cm^–1^ corresponded to the C = O symmetric stretching of COO^–1^ in amino acids. The strong absorption bands at 1263–1265 cm^–1^ belonged to the deformation vibration of C = O in nucleic acids. The bands observed at 1041–1045 cm^–1^ were related to the stretching vibration of polysaccharides. The bands in the region 854–926 cm^–1^ were attributed to the C-C stretching vibration. Overall, the above results showed that proteins and polysaccharides were the main components of EPS. The peak locations of the three samples were similar, indicating that the NaCl stress had no significant effect on the EPS components, and the peaks were shifted slightly, which suggests that the contents of EPS were affected by NaCl stress.

### Effects of NaCl on the Components and Contents of EPS

**FIGURE 7:**
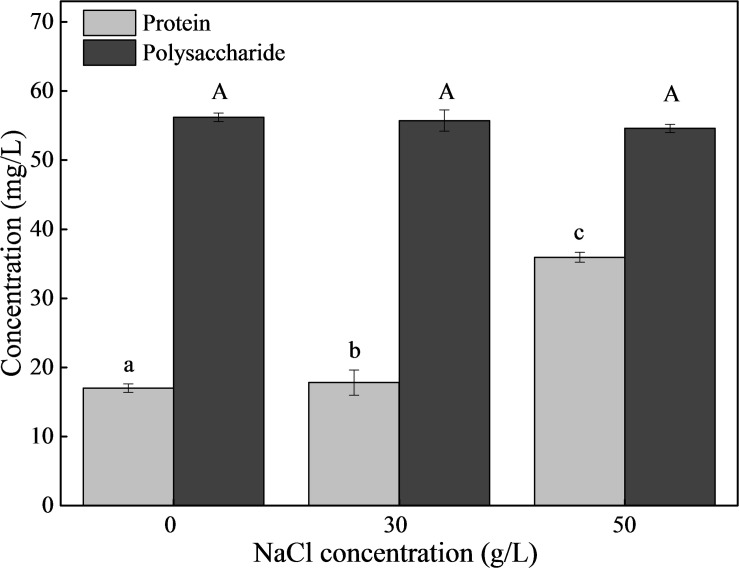
The contents of proteins and polysaccharides in EPS. The strain was collected at logarithmic metaphase under different NaCl stress (0, 30, or 50 g/L), and the EPS was extracted. Different letters indicate the significant differences at *p* < 0.05 (*n* = 3). Values are means ± SD (error bars) for three replicates.

Based on the results of 3D-EEM and FT-IR, the main components of EPS were proteins and polysaccharides. Therefore, we measured the content of proteins and polysaccharides (Figure 7). The concentration of polysaccharides was about 55 mg/L and remained stable under different NaCl stresses. As for proteins, the content was 17.81 mg/L in the absence of NaCl, and increased to 35.93 mg/L with 50 g/L NaCl. In addition, we determined the compositions and contents of protein secondary structures under different NaCl stresses (Table 1). The results showed that NaCl stress had a significant influence on protein secondary structure. In the absence of NaCl, the random coil was the predominant structure, and the relative content decreased from 35.4% (0 g/L NaCl) to 17.7% (50 g/L NaCl) with increasing NaCl content. The β-Sheets dominated the protein secondary structure in the presence of NaCl stress, with a significant increase in content from 15.6% (0 g/L NaCl) to 44.9% (50 g/L NaCl). In addition, both β-turn and α-Helices decreased with increasing NaCl concentrations.

**TABLE 1:** Effect of NaCl on protein secondary structure of EPS.

Type (%)	NaCl content (g/L)		
	
	0	30	50
α-Helices	23.7%	19.4%	16.6%
β-Sheets	15.6%	32.0%	44.9%
β-turn	25.3%	23.1%	20.8%
Random coil	35.4%	25.5%	17.7%

## Discussion

A strain with great denitrifying performance was isolated from high-salinity wastewater, and this salt-tolerant isolate was identified as *Pseudomonas* sp. DN-23. Culture conditions were optimized and the results suggested that the optimum carbon source was sodium acetate, C/N 12:1, pH 8, and a temperature of 30°C. As for the carbon source, sodium acetate is also the optimum carbon source for *Vibrio diabolicus* SF16, which is a halophilic nitrifying-denitrifying bacterium (Duan et al., 2015). As for the C/N, the greatest biomass and denitrification rate of strain DN-23 was obtained at C/N 12:1, similar to other heterotrophic nitrifying-aerobic denitrifying bacteria such as *Pseudomonas stutzeri* YG-24 (Li et al., 2015) and *Agrobacterium* sp. LAD9 (Yu et al., 2015). In terms of pH, our work was consistent with the most reported nitrifying-denitrifying bacteria, such as *Bacillus methylotrophicus* strain L7 (Zhang et al., 2012) and *Acinetobacter junii* YB (Ren et al., 2014), showing better nitrifying-denitrifying abilities at a neutral or alkaline environment. Regarding temperature, most reported nitrifying-denitrifying are mesophiles, such as *Acinetobacter junii* YB (Ren et al., 2014) and *Pseudomonas* sp. C27 (Chen et al., 2013a), and they can barely grow at 4°C.

The denitrification performance of DN-23 under salt stress was determined under optimized conditions (Figure 2), and the results showed that the denitrifying rate decreased with increasing NaCl concentration. The denitrification rate of strain DN-23 was 87.65% under 50 g/L NaCl stress, which was greater than other salt-tolerant bacteria, such as *Achromobacter* sp. GAD-3 (Gui et al., 2017) with an 80% denitrification rate at 50 g/L NaCl and *Bacillus methylotrophicus* strain L7 (Zhang et al., 2012) with a 39% nitrogen removal rate at 40 g/L NaCl. Special attention was paid to the accumulated intermediate nitrite under salt stress, and the nitrite content was rapidly degraded in the middle of the denitrifying process, which may be ascribed to the exhaustion of nitrate and the great denitrifying performance. This was different from *A*. sp. GAD-3, whose accumulated nitrite increased to 87.4 mg/L at the end of the denitrification process under 50 g/L NaCl stress (Gui et al., 2017). Wang et al. (2017) isolated a nitrifying-denitrifying bacterium named *Halomonas* sp. strain B01 from the sediment of a saltern pool. After 180 h, 99.2% inorganic nitrogen was removed by *H*. sp. strain B01, whereas lots of intermediates like nitrate and nitrite accumulated. The findings above suggest that the strain DN-23 has a faster denitrifying rate and fewer accumulated intermediates under high salt conditions, while the formation of intermediates is related to the expression of nitrifying and denitrifying genes. For instance, the gene NiR is responsible for the production of nitrite. It has been reported that denitrifying genes can be used as biomarkers to explore the relationship between the performance of reactors and the expression of denitrifying genes (Kuypers et al., 2018). Deng et al. (2019) also reported that salinity inhibits denitrification gene expression using multiple molecular omics, which could be adopted in our future research.

Previous research revealed that microorganisms secrete excess reactive oxygen species (ROS) under adverse conditions, and ROS destroy normal metabolism by injuring cellular components and enhancing peroxidation. Antioxidant enzymes can neutralize the toxic effects of subproducts of oxygen utilization (Zelko et al., 2002), so we determined the activity of antioxidative enzymes (SOD, POD, CAT). The results showed that the strain DN-23 secreted more antioxidative enzymes in response to NaCl stress. Among them, SOD activity decreased under conditions of 50 g/L NaCl, possibly because high NaCl concentrations injure cells and inhibit antioxidative enzymes (Figure 3). Amaretti et al. (2013) also demonstrated similar phenomena. Resisting the damaging effects of NaCl by POD may be insufficient for maintaining a relatively low concentration. Meanwhile, CAT activity increased with NaCl concentration, possibly indicating that it plays a more important role in NaCl resistance.

In the present study, the auto-aggregation performance of DN-23 was investigated, and it was noteworthy that the strain DN-23 exhibited better auto-aggregation performance in response to NaCl stress (Figure 4A). In addition, quantitative analyses of flocculating efficiency and auto-aggregation rate demonstrated that the auto-aggregation rate of DN-23 under high NaCl stress was greater than that of other reported bacteria, such as *Enterobacter* sp. strain FL (Wang et al., 2018) and *Escherichia coli* (Eboigbodin and Biggs, 2008). To explain why NaCl stress increased the flocculation rate of DN-23, FT-IR and 3D-EEM were used to analyze the EPS components (Figures 5, 7), and the results showed that proteins and polysaccharides were the main components of EPS. Among the FT-IR results, the C = O stretching vibration was related to β-sheets in secondary protein structures, which favored bio-flocculation (Badireddy et al., 2010). Additionally, the bands of C-H stretching, C = O symmetric stretching, and C-C stretching in EPS represent hydrophobic functional groups, which may contribute to the auto-aggregation of cells (Jia et al., 2017). Furthermore, the existence of Tyrosine in EPS also provided more evidence for maintaining the stability of granular structures (Zhu et al., 2012). Similar components of EPS were also found in *Enterobacter* sp. strain FL (Wang et al., 2018) and *Bacillus megaterium* TF10 (Sun et al., 2012). In addition to pure strains, biofilm activated sludge and granular sludge also have similar EPS components (Liang et al., 2010; Corsino et al., 2019). Furthermore, the fluorescent intensities of EPS increased with NaCl stress, indicating that NaCl stress affected the secretion of extracellular proteins, which may be related to the enhancement of auto-aggregation of cells cultivated in DM with greater NaCl concentrations (Figures 4, 5). It has been proven that protein-like substances play a crucial role in forming and stabilizing the structure of aggregates (Zhu et al., 2015).

Based on the results of FT-IR and EEM, the content of proteins and polysaccharides was determined (Figure 7). Although some researchers have explored the effects of NaCl stress on the nitrogen removal performance and EPS characteristics of activated sludge, the effects of NaCl stress on the EPS compositions of salt-tolerant denitrifying bacteria have rarely been reported (Campo et al., 2018; Corsino et al., 2018). In the present study, quantitative analysis was used to explore the EPS characteristics of DN-23 under NaCl stress. NaCl stress had little effect on the polysaccharide concentration of EPS, while a significant increase in protein concentration was observed during salt stress. Compared to what was described in the literature (Yan et al., 2013; Xu et al., 2014), the protein content of EPS was much higher, which may be due to the presence of a number of exoenzymes, as suggested by Frølund et al. (1995). Additionally, the protein content increased under NaCl stress, which may result from cells producing more exoenzymes when cultivated in an unfavorable environment (Adav and Lee, 2008). It is noteworthy that the variation of protein content under NaCl stress was in accordance with the variation of auto-aggregation ability, indicating that proteins rather than polysaccharides played a more important role in auto-aggregation ability. Similarly, Adav and Lee (Adav and Lee, 2008) have reported that the auto-aggregation of *Acinetobacter calcoaceticus*, cultivated in media with different phenol concentrations, was significantly stimulated by phenol. The auto-aggregation of *A. calcoaceticus* was positively correlated with protein content, and the secreted proteins contributed to the formation of mature granules. Wang et al. (2018) also suggested that proteins in the EPS secreted by *Enterobacter* sp. strain FL were important for the flocculation and aggregation of microbes.

The distribution of each protein’s secondary structures was measured (Table 1), and the results showed that NaCl stress significantly affected the protein secondary structures of EPS. Damodaran and Kinsella reported similar results, suggesting that NaCl affects the hydrophobicity and hydrophilicity of proteins by altering the protein conformational structures. Zhao et al. (2011) also found that salt influences the ellipticity values of proteins near 190 nm according to the CD spectra. Additionally, certain protein secondary structures, such as β-Sheets and α-Helices, promote bioflocculation, while random coils and antiparallel β-sheets decrease bioflocculation ability (Omoike and Chorover, 2004). In the present study, β-Sheets, which accounted for the majority of secondary structures, increased significantly with increasing NaCl concentrations, which may be related to the increased auto-aggregation rate in response to greater NaCl stress. The relative content of each protein secondary structure was different from that described in other reports. In *Enterobacter* sp. Strain FL, the random coil secondary structure accounted for 40% (Wang et al., 2018). Badireddy et al. (2010) found that the random coil in EPS from activated sludge was predominant during the exponential phase. Based on the above findings, we concluded that NaCl stress alters the protein secondary structures of EPS and increases the auto-aggregation ability of the strain TN-10.

In summary, a salt-tolerant denitrifying bacterium was isolated and identified, and this strain was able to remove 86.38% of nitrate without nitrite accumulation under 50 g/L NaCl. Additionally, the isolate secreted more antioxidative enzymes in response to NaCl stress. It is worth noting that the strain exhibited greater auto-aggregation ability under NaCl stress. Furthermore, proteins and polysaccharides were the main components of EPS, and it was proteins rather than polysaccharides that played a more important role in auto-aggregation. The protein secondary structures of EPS were altered under NaCl stress, which may be related to the change in auto-aggregation.

## Data Availability Statement

The raw data supporting the conclusions of this article will be made available by the authors, without undue reservation, to any qualified researcher.

## Author Contributions

DL and CW designed the study and analyzed the data. DL and XL performed the experiments. DL wrote the manuscript. CW revised the manuscript.

## Conflict of Interest

The authors declare that the research was conducted in the absence of any commercial or financial relationships that could be construed as a potential conflict of interest.

## Footnotes

1 https://www.ncbi.nlm.nih.gov/
