# Supplementary material for: Characteristics of Nitrogen Removal and Extracellular Polymeric Substances of a Novel Salt-Tolerant Denitrifying Bacterium, Pseudomonas sp. DN-23
Source: Front Microbiol. 2020 Mar 6;11:335. doi: 10.3389/fmicb.2020.00335 (PMC7067702; doi:10.3389/fmicb.2020.00335)
Supplement: Supplementary file 1 [file Image_1.pdf]

Characteristics of nitrogen removal and extracellular polymeric substances of a novel salt-tolerant denitrifying bacterium, *Pseudomonas* sp. DN-23

Dan Li[a,b], Xihong Liang[a,b], Chongde Wu[a,b,*]

[a]College of Biomass Science and Engineering, Sichuan University, Chengdu 610065, China. [b]Key Laboratory of Leather Chemistry and Engineering, Ministry of Education, Sichuan University, Chengdu 610065, China.

***Corresponding author:**

Chongde Wu

Mailing address: College of Biomass Science and Engineering, Sichuan University, Chengdu 610065, China

Phone: +86-28-85406149, Fax: +86- 28-85405237

E-mail: cdwu@scu.edu.cn

Fig. S1 Phylogenetic tree based on the 16S rDNA sequence of strain DN-23 and other reference sequences. The numbers at the forks indicate the bootstrap values and >50% bootstrap values are shown at the branch points. Bar indicates the nucleotide difference per sequence position.

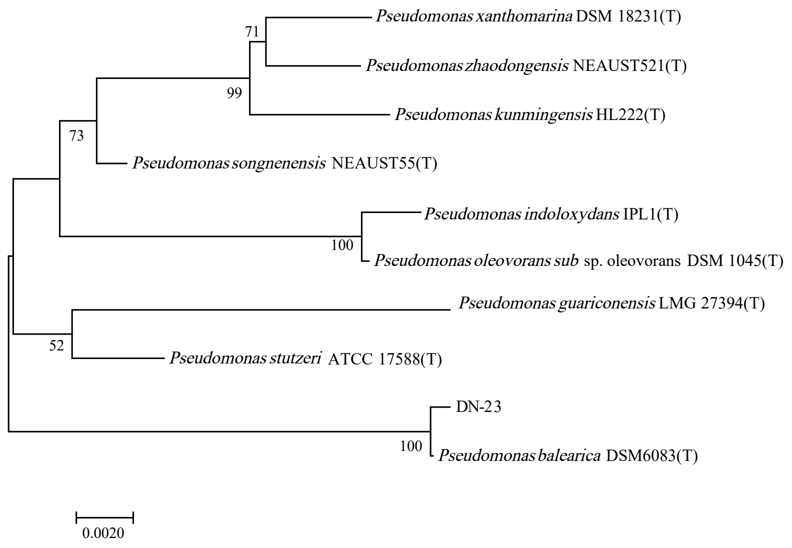

Fig. S1
